# Evaluating AI performance in infectious disease education: a comparative analysis of ChatGPT, Google Bard, Perplexity AI, Microsoft Copilot, and Meta AI

**DOI:** 10.3389/fmed.2025.1679153

**Published:** 2025-10-13

**Authors:** Abdulaziz Ibrahim Alzarea, Azfar Athar Ishaqui, Muhammad Bilal Maqsood, Abdullah Salah Alanazi, Aseel Awad Alsaidan, Tauqeer Hussain Mallhi, Narendar Kumar, Muhammad Imran, Sultan M. Alshahrani, Hassan H. Alhassan, Sami I. Alzarea, Omar Awad Alsaidan

**Affiliations:** ^1^Department of Clinical Pharmacy, College of Pharmacy, Jouf University, Sakaka, Al-Jouf, Saudi Arabia; ^2^Department of Clinical Pharmacy, College of Pharmacy, King Khalid University, Abha, Saudi Arabia; ^3^Eastern Health Cluster, Ministry of Health, Dammam, Saudi Arabia; ^4^Department of Family and Community Medicine, College of Medicine, Jouf University, Sakaka, Al-Jouf, Saudi Arabia; ^5^Medicines R Us Chemist, Gregory Hills, NSW, Australia; ^6^School of Pharmacy, Faculty of Health and Medical Sciences, Taylors University, Selangor, Malaysia; ^7^Department of Pharmacy Practice, Faculty of Pharmacy, Sindh University, Jamshoro, Pakistan; ^8^Department of Pharmacy, Iqra University, Karachi, Pakistan; ^9^Department of Clinical Laboratory Sciences, College of Applied Medical Sciences, Jouf University, Sakaka, Al-Jouf, Saudi Arabia; ^10^Department of Pharmacology, College of Pharmacy, Jouf University, Sakaka, Al-Jouf, Saudi Arabia; ^11^Department of Pharmaceutics, College of Pharmacy, Jouf University, Sakaka, Al-Jouf, Saudi Arabia

**Keywords:** infectious disease, artificial intelligence, ChatGPT, Google Bard, Perplexity AI, Microsoft Copilot, Meta AI

## Abstract

**Background:**

This study systematically evaluates and compares the performance of ChatGPT 3. 5, Google Bard (Gemini), Perplexity AI, Microsoft Copilot, and Meta AI in responding to infectious disease-related multiple-choice questions (MCQs).

**Methods:**

A systematic comparative study was conducted using 20 infectious disease case studies sourced from Infectious Diseases: A Case Study Approach by Jonathan C. Cho. Each case study included 7–10 MCQs, resulting in a total of 160 questions. AI platforms were provided with standardized prompts containing the case study text and MCQs without additional context. Their responses were evaluated against a reference answer key from the textbook. Accuracy was measured by the percentage of correct responses, and consistency was assessed by submitting identical prompts 24 h apart.

**Results:**

ChatGPT 3.5 achieved the highest numerical accuracy (65.6%), followed by Perplexity AI (63.2%), Microsoft Copilot (60.9%), Meta AI (60.8%), and Google Bard (58.8%). AI models performed best in symptom identification (76.5%) and worst in therapy-related questions (57.1%). ChatGPT 3.5 demonstrated strong diagnostic accuracy (79.1%) but had a significant drop in antimicrobial treatment recommendations (56.6%). Google Bard performed inconsistently in microorganism identification (61.9%) and preventive therapy (62.5%). Microsoft Copilot exhibited the most stable responses across repeated testing, while ChatGPT 3.5 showed a 7.5% accuracy decline. Perplexity AI and Meta AI struggled with individualized treatment recommendations, showing variability in drug selection and dosing adjustments. AI-generated responses were found to change over time, with some models giving different antimicrobial recommendations for the same case scenario upon repeated testing.

**Conclusion:**

AI platforms offer potential in infectious disease education but demonstrate limitations in pharmacotherapy decision-making, particularly in antimicrobial selection and dosing accuracy. ChatGPT 3.5 performed best but lacked response stability, while Microsoft Copilot showed greater consistency but lacked nuanced therapeutic reasoning. Further research is needed to improve AI-driven decision support systems for medical education and clinical applications through clinical trials, evaluation of real-world patient data, and assessment of long-term stability.

## 1 Introduction

Medical education, together with clinical decision-making, has experienced a transformation through the adoption of artificial intelligence (AI). Medical organizations use AI-driven models to enhance their diagnostic capabilities as well as treatment recommendation systems while providing training for healthcare professionals. The AI-driven models create new ways to disseminate knowledge while improving decision support systems (1). The implementation of AI-based Clinical Decision Support Systems helps medical personnel analyze disease distribution patterns while improving their ability to make accurate diagnoses (2). Medical professionals continue to have doubts about the reliability of AI response systems that operate in advanced medical conditions (3).

Medical education has recently seen increased interest in the application of AI in its practice. Multiple recent investigations demonstrate how AI enhances learning outcomes as well as clinical reasoning performance (4). The ability of medical students and professionals to understand disease processes and optimize treatment decisions is being investigated through AI-driven models, including ChatGPT, Google Bard (Gemini), Perplexity AI, Microsoft Copilot, and Meta AI (5). There is a lack of comprehensive research that evaluates the performance of AI platforms in handling standardized medical case-based questions (6). The application of AI-generated content in medical education requires careful analysis to ensure reliability and clinical relevance (7).

The diagnostic methods for infectious diseases, along with the complex requirements for antimicrobial treatment, create specific barriers for medical staff. The continuous updates to AI models raise doubts about their ability to consistently generate the same responses in the future. AI has become increasingly important for infectious disease education and clinical decision-making because it supports diagnosis, treatment recommendations, and pharmacotherapy management (8). The reliability and consistency of AI systems in managing infectious diseases require assessment because they affect both clinical education and decision support systems (9).

Medical education experts debate the potential of large language AI models such as ChatGPT, Google Bard, Perplexity AI, Microsoft Copilot and Meta AI because their clinical applications produce inconsistent and unreliable results (10). Recent assessment reports show that AI systems produce substantial errors when used across various medical practice domains. The neurosurgical board examination success rate for ChatGPT-4 reached 82.6% according to Ali et al. (11) but the model failed to answer 56.6% of infectious disease-related case-based multiple-choice questions (MCQs) as per Chaves Fernandes et al. (12). Experts have identified Perplexity AI as easily readable and understandable yet questions persist about its ability to produce validated pharmacotherapy recommendations (13). The medical practice of treating infections using AI-produced treatment recommendations proved to have inadequate accuracy in both medication selection and dosage precision according to Langford et al. (14). Research shows that AI generates different responses because various models produce different treatment plans for the same clinical cases when tested multiple times (15).

The handling of clinical situations and medical educational inquiries by AI models has been studied extensively. The diagnostic and information retrieval functions of ChatGPT demonstrate strength, yet the system encounters difficulties in maintaining up-to-date content (16). Real-time internet data processing by Google Bard has shown effectiveness, although its occasionally unreliable responses raise concerns about reliability (17). Perplexity AI demonstrates outstanding readability and exceptional comprehension capabilities in medical implementation (18). The clinical guidance features of Copilot have been validated in dermatological and surgical queries (19), but additional evaluation is required to determine its reliability compared to human expertise. The medical knowledge retrieval capabilities of Meta AI exist, yet sufficient evidence for healthcare implementation has not been established. Evaluation protocols must assess both the accuracy of AI-generated health information and its ability to consistently reproduce results when used for infectious disease education and antimicrobial prescribing. Additional research should directly compare AI-generated medical information to assessments made by human experts because infectious disease instruction and antimicrobial treatment depend on high accuracy and reliability.

The research fails to provide essential information about the performance outcomes of different AI systems in infectious disease detection and pharmacotherapy development. Research about AI mostly examines individual models while providing limited thorough assessments of multiple platforms when dealing with antimicrobial stewardship and infectious disease treatment choices. To achieve clinical reliability targets, recent advancements in AI require a method to determine response reproducibility. The study aims to determine how well ChatGPT, Google Bard (Gemini), Perplexity AI, Microsoft Copilot, and Meta AI perform in answering pharmacotherapy questions related to infectious diseases to determine their usability in medical education and practice.

## 2 Material and methods

### 2.1 Study design

In order to compare five of the most popular AI tools, including ChatGPT 3.5, Google Bard (Gemini), Perplexity AI, Microsoft Copilot, and Meta AI, the MCQs about the cases of infectious diseases were asked from these AI tools during months February and March 2025. The case study method, AI prompts developed for the purpose of the research, data collection, and comparison of the results were used as the research methods.

### 2.2 Selection of AI models for clinical evaluation

The purpose of this research was to evaluate the educational and clinical performance capacities of the chosen artificial intelligence models. Concretely, it compared the performance of ChatGPT 3.5 (GPT-3.5 Turbo), Google Bard (Gemini 1.5 Pro), Perplexity AI, Microsoft Copilot (powered by GPT-4 Turbo), and Meta AI (LLaMA-based model) in clinical scenario and medical education questions, using versions available in February–March 2025. ChatGPT 3.5 and Meta AI relied on pre-trained data, while Google Bard, Perplexity AI, and Microsoft Copilot incorporated real-time data access or continuous updates.

### 2.3 Case study and source material selection

The case studies used in this research were obtained from the book Infectious Diseases: A Case Study Approach by Jonathan C. Cho, McGraw-Hill Education (2020). In the case studies, clinical scenarios entail the students to assess the pharmacological management of infectious diseases. It provides a lot of information about various bacterial, viral, fungal and parasitic infections, including the most typical and atypical ones. The purpose of this paper is to demonstrate the correct approach in the management of infections through medication. All the case studies are accompanied by multiple-choice comprehension questions that the students can answer on the content and the correct answers for all the options are provided.

The case studies in “Infectious Diseases: A Case Study Approach” contain comprehensive patient data that include the chief complaint and past medical history of the diseases and the previous surgeries and current medications and smoking status and family medical history. The assessment includes vital sign checks and targeted examination results for the main complaint along with test results such as blood tests and cultures and imaging outcomes. The cases present diagnostic and therapeutic content together with MCQs for assessing both knowledge understanding and clinical decision-making abilities.

### 2.4 MCQ extraction and preparation

The textbook includes 20 case studies, each containing 7–10 MCQs that evaluate students' understanding of both case content and infectious disease principles. Each case study contained all its corresponding MCQs used in this research. Researchers extracted the complete case study text along with its MCQs (including all question stems and answer options) directly from the textbook. The AI platforms accessed the same information that a human reader would see when using the textbook.

### 2.5 Standardized AI prompting

All AI platforms received their input through a uniform prompt structure to ensure consistent and clear processing. Each complete case study text was provided as a single input containing the MCQ question stem and all answer options (A through E) as they appeared in the textbook. The AI was instructed to choose one correct response from the available options for each MCQ in the prompt. The assessment included only the information directly from the case studies and MCQs, without supplementary instructions that would mimic typical textbook usage for students. The same prompt structure was used as the basis for all AI systems. The original material from the book remained unaltered in this assessment.

### 2.6 Categorization of cases

**Figure 1:**
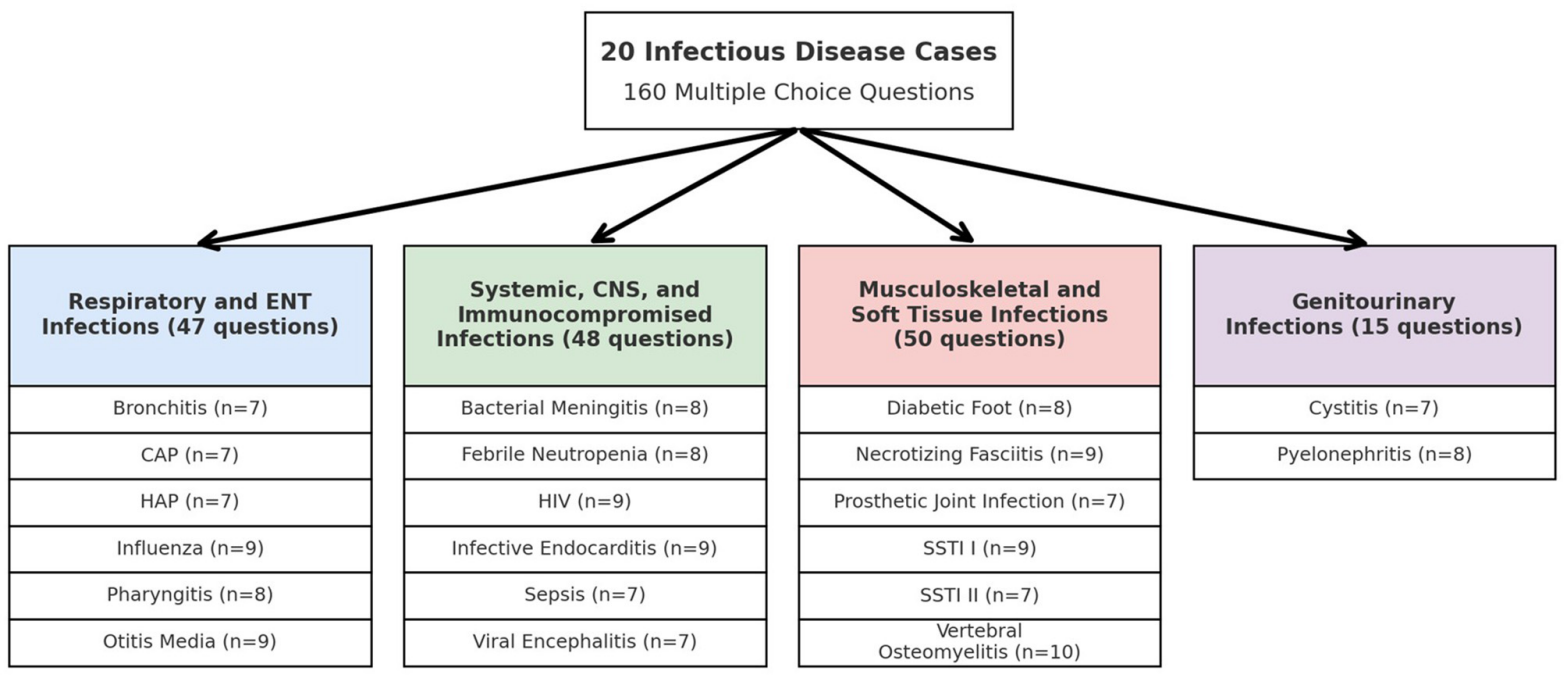
Flowchart distribution of 160 multiple choice questions across 20 infectious disease cases.

This research examined 20 case studies, which included four main infection groups: respiratory and ENT infections; systemic, central nervous system, and immunocompromised infections; musculoskeletal and soft tissue infections; and genitourinary infections. Figure 1 illustrates the distribution of all 20 case studies and their associated MCQs across these infection groups in a flowchart. MCQs assessments were developed to evaluate diagnostic skills as well as treatment choices in each case, spanning a total of 160 questions. The same set of MCQs was presented to ChatGPT 3.5, Perplexity AI, Google Bard (Gemini), Microsoft Edge Copilot, and Meta AI for diagnostic accuracy assessment. The evaluation of each model focused on their correct and incorrect responses to determine their clinical reasoning abilities across different infectious disease cases.

### 2.7 Case study content and structure

The case studies in “Infectious diseases: a case study approach” present complete clinical situations that feature patients' chief complaints, comprehensive medical histories, and social aspects, including smoking behavior and family medical background. The assessment includes vital sign measurements, physical examination results, and laboratory findings related to the presenting problem, as well as blood tests, cultures, and imaging results. The case provides diagnostic and treatment information, including MCQs that evaluate students' understanding and clinical judgment skills.

### 2.8 Answer validation and comparison

- Reference answer key: the correct answers to all MCQs were derived from the book itself. These answers served as the gold standard for evaluating AI-generated responses.
- Response matching: each AI platform's answers were systematically compared against the reference answer key.
- Scoring criteria: correct responses were assigned a score of 1. While, Incorrect responses were assigned a score of 0. Partial credit was not awarded.

### 2.9 Quality assurance

Independent reviewers conducted double confirmation of all data inputs, AI outputs, and manual evaluations. Multiple checks were established throughout the data collection process and analysis phase to reduce human error.

### 2.10 Response consistency and reproducibility assessment

The stability and reliability of AI platforms were assessed through two identical prompt submissions to each system—one at the beginning and one after a 24-h waiting period. Each case study received identical inputs twice during both sessions, while researchers recorded the resulting outputs. Comparisons were conducted both within each system to verify temporal consistency and between systems to establish overall reproducibility. The predefined scoring system indicated that responses were considered highly consistent when they matched the reference answer key in both testing periods.

### 2.11 Statistical analysis

The researcher used SPSS software to perform the statistical analysis. Descriptive statistics were used to calculate the mean, standard deviation, median, and range for the performance summary. The accuracy assessment involved comparing AI-generated responses to the reference answer keys, while the performance evaluation was conducted across different infectious disease categories and clinical knowledge domains, including diagnosis, microorganism identification, therapy, and preventive strategies. The testing of AI models with duplicate questions occurred twice within a 24-h interval to determine response consistency by recording variations. The comparison of AI model accuracy relied on chi-square or Fisher's exact tests, depending on the situation, with a statistical significance threshold of *p* < 0.05. Odds ratios and their 95% confidence intervals were calculated to assess effect sizes and enhance comparability of AI performance across clinical question types. Non-significant results (*p* ≥ 0.05) are reported with exact p-values where applicable.

## 3 Results

A total of 160 MCQs from infectious disease case studies were used to measure the accuracy of five AI platforms, including ChatGPT 3.5, Perplexity AI, Google Bard (Gemini), Microsoft Edge Copilot, and Meta AI. It also evaluated ChatGPT as the most reliable AI platform with 65.6% accuracy and 34.4% incorrect responses. ChatGPT 3.5 was similar to Perplexity AI in accuracy at 63.2%, while Google Bard (Gemini) had the lowest accuracy at 58.8% and the highest rate of incorrect answers at 41.2%. However, Microsoft Edge Copilot and Meta AI had comparable accuracy rates (60.9 and 60.8 percent respectively). Different case studies show inconsistent results from the systems, which have varying performance levels across all models as indicated by the standard deviations. Moreover, the range of correct answers (14.3%−100%) indicates that each AI had some cases with high and some with low performance. The overall performance statistics are summarized in Table 1.

**Table 1:** Overall accuracy of AI platforms on infectious disease case studies MCQs (*N* = 160).

	**Overall correct answers summarize statistic**					**Overall wrong answers summarize statistic**				
	***N***	**Average**	**SD**	**Median**	**Range**	***N***	**Average**	**SD**	**Median**	**Range**
CHAT GPT	105	65.6%	23.2%	70.7%	14.3%−100%	55	34.4%	23.2%	29.3%	0%−85.7%
Perplexity AI	101	63.2%	23.6%	69.1%	14.3%−100%	59	36.9%	23.6%	31%	0%−85.7%
Google Bard (Gemini)	94	58.8%	23.3%	61.3%	14.3%−100%	66	41.2%	23.3%	38.8%	0%−85.7%
Microsoft edge COPILOT	97	60.9%	28.4%	64.6%	14.3 %−100%	63	39.1%	28.4%	35.4%	0%−85.7%
Meta AI	96	60.8%	24.6%	66.7%	20%−85.7%	64	39.2%	21.6%	33.3%	14.2%−80%

### 3.1 Case-by-case performance of AI platforms on MCQs from infectious disease case studies

Five AI platforms (ChatGPT 3.5, Perplexity AI, Google Bard (Gemini), Microsoft Edge Copilot, and Meta AI) were applied to 20 different infectious disease cases, whose performance is presented in Table 2. The AI platforms demonstrated varying levels of accuracy across different infectious disease case studies, with ChatGPT 3.5 emerging as the most consistent performer, achieving a 100% accuracy rate in Bronchitis and Community-Acquired Pneumonia, and scoring high in Febrile Neutropenia (75%), HIV (77.8%), and Diabetic Foot (87.5%). Perplexity AI followed closely, showing strong performance in Diabetic Foot (100%) and Skin Soft Tissue Infection II (100%), but struggled in Pyelonephritis (37.5%) and Viral Encephalitis (28.6%). Google Bard (Gemini) had the weakest overall performance, with low accuracy in Infective Endocarditis (22.2%), Viral Encephalitis (14.3%), and Cystitis (28.6%). Microsoft Edge Copilot showed strong results in Sepsis (100%) and Febrile Neutropenia (100%), but exhibited inconsistencies across cases such as Hospital-Acquired Pneumonia (14.3%) and Pyelonephritis (37.5%). Meta AI maintained moderate accuracy, performing well in Sepsis (85.7%) and Skin Soft Tissue Infection I (85.7%), but struggled in Vertebral Osteomyelitis (20%) and Cystitis (28.6%).

**Table 2:** Case-by-case performance of AI platforms on MCQs from infectious disease case studies.

**Diagnosis**	**No. of questions**	**ChatGPT**		**Perplexity AI**		**Google Bard (Gemini)**		**Microsoft edge COPILOT**		**Meta AI**	
		**Correct answer** ***N*** **(%)**	**Wrong answers** ***N*** **(%)**	**Correct answer** ***N*** **(%)**	**Wrong answers** ***N*** **(%)**	**Correct answer** ***N*** **(%)**	**Wrong answers** ***N*** **(%)**	**Correct answer** ***N*** **(%)**	**Wrong answers** ***N*** **(%)**	**Correct answer** ***N*** **(%)**	**Wrong answers** ***N*** **(%)**
**Respiratory and ENT infections**											
Bronchitis	7	7 (100)	0 (0)	6 (85.7)	1 (14.3)	6 (85.7)	1 (14.3)	7 (100)	0 (0)	6 (85.7)	1 (14.3)
Community acquired pneumonia	7	7 (100)	0 (0)	5 (71.4)	2 (28.6)	4 (57.1)	3 (42.9)	6 (85.7)	1 (14.3)	5 (71.4)	2 (28.6)
Hospital acquired pneumonia	7	3 (42.9)	4 (57.1)	3 (42.9)	4 (57.1)	2 (28.6)	5 (71.4)	1 (14.3)	6 (85.7)	4 (57.1)	3 (42.9)
Influenza	9	6 (66.7)	3 (33.3)	5 (55.6)	4 (44.4)	4 (44.4)	5 (55.6)	4 (44.4)	5 (55.6)	5 (55.6)	4 (44.4)
Pharyngitis	8	4 (50)	4 (50)	6 (75)	2 (25)	4 (50)	4 (50)	3 (37.5)	5 (62.5)	4 (50)	4 (50)
OTITIS media	9	5 (55.6)	4 (44.4)	6 (66.7)	3 (33.3)	6 (66.7)	3 (33.3)	5 (55.6)	4 (44.4)	6 (66.7)	3 (33.3)
**Systemic, central nervous system, and immunocompromised infections**											
Bacterial meningitis	8	6 (75)	2 (25)	5 (62.5)	3 (37.5)	4 (50)	4 (50)	5 (62.5)	3 (37.5)	5 (62.5)	3 (37.5)
Febrile neutropenia	8	6 (75)	2 (25)	6 (75)	2 (25)	7 (87.5)	1 (12.5)	8 (100)	0 (0)	6 (75)	2 (25)
HIV	9	7 (77.8)	2 (22.2)	6 (66.7)	3 (33.3)	6 (66.7)	3 (33.3)	6 (66.7)	3 (33.3)	6 (66.7)	3 (33.3)
Infective endocarditis	9	2 (22.2)	7 (77.8)	2 (22.2)	7 (77.8)	2 (22.2)	7 (77.8)	2 (22.2)	7 (77.8)	2 (22.2)	7 (77.8)
Sepsis	7	5 (71.4)	2 (28.6)	5 (71.4)	2 (28.6)	7 (100)	0 (0)	7 (100)	0 (0)	6 (85.7)	1 (14.3)
Viral encephalitis	7	3 (42.9)	4 (57.1)	2 (28.6)	5 (71.4)	1 (14.3)	6 (85.7)	2 (28.6)	5 (71.4)	2 (28.6)	5 (71.4)
**Musculoskeletal and soft tissue infections**											
Diabetic foot	8	7 (87.5)	1 (12.5)	8 (100)	0 (0)	5 (62.5)	3 (37.5)	7 (87.5)	1 (12.5)	6 (75)	2 (25)
Necrotizing fasciitis	9	8 (88.9)	1 (11.1)	7 (77.8)	2 (22.2)	6 (66.7)	3 (33.3)	7 (77.8)	2 (22.2)	7 (77.8)	2 (22.2)
prosthetic joint infection	7	5 (71.4)	2 (28.6)	5 (71.4)	2 (28.6)	6 (85.7)	1 (14.3)	5 (71.4)	2 (28.6)	6 (85.7)	1 (14.3)
Skin soft tissue infection I	9	5 (55.6)	4 (44.4)	7 (77.8)	2 (22.2)	7 (77.8)	2 (22.2)	6 (66.7)	3 (33.3)	6 (66.7)	3 (33.3)
Skin soft tissue infection II	7	6 (85.7)	1 (14.3)	7 (100)	0 (0)	5 (71.4)	2 (28.6)	6 (85.7)	1 (14.3)	6 (85.7)	1 (14.3)
Vertebral osteomyelitis	10	7 (70)	3 (30)	6 (60)	4 (40)	6 (60)	4 (40)	6 (60)	4 (40)	2 (20)	8 (80)
**Genitourinary infections**											
Cystitis	7	1 (14.3)	6 (85.7)	1 (14.2)	6 (85.7)	2 (28.6)	5 (71.4)	1 (14.3)	6 (85.7)	2 (28.6)	5 (71.4)
Pyelonephritis	8	5 (62.5)	3 (37.5)	3 (37.5)	5 (62.5)	4 (50)	4 (50)	3 (37.5)	5 (62.5)	4 (50)	4 (50)

### 3.2 Performance analysis of AI response accuracy across clinical content domains

Table 3 presents the performance of the AI platforms across distinct clinical question domains. In the “Symptoms” category, ChatGPT 3.5, Perplexity AI, Microsoft Edge Copilot, and Meta AI each achieved 76.5% correct responses, while Google Bard scored slightly lower at 70.6%. For “Diagnosis” questions, ChatGPT led with 79.1% accuracy, followed by Perplexity AI at 69%, with both Google Bard and Copilot at 62.1% and Meta AI at 58.6%. In the “Microorganism” domain, all platforms exhibited similar performance, with correct answer rates ranging from 61.9% to 66.7%. Regarding “Therapy” questions, ChatGPT 3.5 and Copilot reached 57.1% accuracy, whereas the remaining platforms performed in the low-to-mid 50% range. For “Preventive Therapy,” ChatGPT and Perplexity AI both attained 75% accuracy, contrasting with Meta AI's 50%. Notably, in the “Risk Factor” category, Meta AI excelled with an 87.5% success rate compared to 75% or lower for the other models. Lastly, the “Other” category showed moderate outcomes, with Perplexity AI at 64.3% and the remaining platforms at 50% correct.

**Table 3:** Performance analysis of AI response accuracy across clinical content domains.

**Questions categorization by clinical domain**	**CHAT GPT**		**Perplexity AI**		**Google Bard (Gemini)**		**Microsoft edge COPILOT**		**Meta AI**	
	**Correct answer** ***N*** **(%)**	**Wrong answer** ***N*** **(%)**	**Correct answer** ***N*** **(%)**	**Wrong answer** ***N*** **(%)**	**Correct answer** ***N*** **(%)**	**Wrong answer** ***N*** **(%)**	**Correct answer** ***N*** **(%)**	**Wrong answer** ***N*** **(%)**	**Correct answer** ***N*** **(%)**	**Wrong answer** ***N*** **(%)**
Symptoms (*N* = 17)	13 (76.5)	4 (23.5)	13 (76.5)	4 (23.5)	12 (70.6)	5 (29.4)	13 (76.5)	4 (23.5)	13 (76.5)	4 (23.5)
Diagnosis (*N* = 29)	23 (79.11)	6 (20.7)	20 (69)	9 (31)	18 (62.1)	11 (37.9)	18 (62.1)	11 (37.9)	17 (58.6)	12 (41.4)
Microorganism (*N* = 21)	14 (66.7)	7 (33.3)	14 (66.7)	7 (33.3)	13 (61.9)	8 (38.0)	13 (61.9)	8 (38.1)	13 (61.9)	8 (38.1)
Therapy (*N* = 63)	36 (57.1)	27 (42.9)	33 (52.4)	30 (47.6)	34 (53.9)	29 (46.0)	36 (57.1)	27 (42.8)	35 (55.6)	28 (44.4)
Preventive therapy (*N* = 8)	6 (75)	2 (25)	6 (75)	2 (25)	5 (62.5)	3 (37.5)	5 (62.5)	3 (37.5)	4 (50)	4 (50)
Risk factor (*N* = 8)	6 (75)	2 (25)	6 (75)	2 (25)	5 (62.5)	3 (37.5)	5 (62.5)	3 (37.5)	7 (87.5)	1 (12.5)
Other (*N* = 14)	7 (50)	7 (50)	9 (64.3)	5 (35.7)	7 (50)	7 (50)	7 (50.0)	7 (50)	7 (50)	7 (50)

### 3.3 Consistency of AI platform responses

Table 4 evaluates the reproducibility of AI responses by comparing the number and percentage of correct answers from two rounds of identical prompts, submitted 24 h apart, across 20 infection cases (160 questions in total). ChatGPT 3.5 showed notable variability, with a decrease in accuracy in Bacterial Meningitis (75%−50%), Necrotizing Fasciitis (88.9%−66.7%), and Skin Soft Tissue Infection II (85.7%−57.1%). Perplexity AI demonstrated more stability, maintaining consistent performance in 13 of 20 cases, but saw a drop in Pyelonephritis (37.5%−87.5%) and Sepsis (71.4%−57.1%). Google Bard (Gemini) remained largely inconsistent, with Hospital-Acquired Pneumonia (28.6%) and Pharyngitis (50%) fluctuating significantly, though some cases saw improvement. Microsoft Edge Copilot displayed strong reliability, showing no change in most cases and only minor deviations in responses. Meta AI exhibited the highest instability, with large fluctuations in Vertebral Osteomyelitis (60%−30%) and Skin Soft Tissue Infection II (85.7%−57.1%), suggesting inconsistency in knowledge retention and response generation. These findings indicate that while some AI models maintain their answers over time, others show substantial variability, impacting their reliability for medical education and decision-making.

**Table 4:** Consistency of AI responses to identical infectious disease case study MCQs second time.

**Infection name**	**No. of Qs**	**CHAT GPT**		**Perplexity AI**		**Google Bard (Gemini)**		**Microsoft edge COPILOT**		**Meta AI**	
		**1st vs. 2nd round correct answers**	**Diff**.	**1st vs. 2nd round correct answers**	**Diff**.	**1st vs. 2nd round correct answers**	**Diff**.	**1st vs. 2nd round correct answers**	**Diff**.	**1st vs. 2nd round correct answers**	**Diff**.
Overall	160	105 (65.6%) vs. 93 (58.1%)	−7.50%	101 (63.1%) vs. 100 (62.5%)	−0.60%	94 (58.8%) vs. 93 (58.1%)	−0.70%	97 (60.6%) vs. 97 (60.6%)	0%	96 (60%) vs. 97 (60.6%)	0.60%
**Mild infections**											
Bronchitis	7	7 (100%) vs. 7 (100%)	0%	6 (85.7%) vs. 6 (85.7%)	0%	6 (85.7%) vs. 7 (100%)	−14.3%	7 (100%) vs. 6 (85.7%)	14.3%	6 (85.7%) vs. 6 (85.7%)	0%
Cystitis	7	1 (14.3%) vs. 2 (28.6%)	−14.30%	1 (14.3%) vs. 1 (14.3%)	−14.3%	2 (28.6%) vs. 0 (0%)	28.6%	1 (14.3%) vs. 0 (0%)	14.3%	2 (28.6%) vs. 2 (28.6%)	0%
Influenza	9	6 (66.7%) vs. 6 (66.7%)	0%	5 (55.6%) vs. 5 (55.6%)	0%	4 (44.4%) vs. 4 (44.4%)	0%	4 (44.4%) vs. 4 (44.4%)	0%	5 (55.6%) vs. 5 (55.6%)	0%
Otitis media	9	5 (55.6%) vs. 3 (33.3%)	22.20%	6 (66.7%) vs. 6 (66.7%)	22.2%	6 (66.7%) vs. 5 (55.6%)	11.1%	5 (55.6%) vs. 8 (88.9%)	−33.3%	6 (66.7%) vs. 5 (55.6%)	11.1%
Pharyngitis	8	4 (50%) vs. 4 (50%)	0%	6 (75%) vs. 5 (62.5%)	0%	4 (50%) vs. 5 (62.5%)	−12.5%	3 (37.5%) vs. 7 (87.5%)	−50%	4 (50%) vs. 3 (37.5%)	12.5%
Skin soft tissue infection I	9	5 (55.6%) vs. 5 (55.6%)	0%	7 (77.8%) vs. 6 (66.7%)	0%	7 (77.8%) vs. 5 (55.6%)	22.2%	6 (66.7%) vs. 6 (66.7%)	0%	6 (66.7%) vs. 5 (55.6%)	11.1%
**Moderate infections**											
Community acquired pneumonia	7	7 (100%) vs. 6 (85.7%)	14.00%	5 (71.4%) vs. 5 (71.4%)	14.3%	4 (57.1%) vs. 5 (71.4%)	−14.3%	6 (85.7%) vs. 7 (100%)	−14.23%	5 (71.4%) vs. 6 (85.7%)	−14.3%
Diabetic foot	8	7 (87.5%) vs. 7 (87.5%)	0%	8 (100%) vs. 7 (87.5%)	0%	5 (62.5%) vs. 7 (87.5%)	−25%	7 (87.5%) vs. 5 (62.5%)	25%	6 (75%) vs. 6 (75%)	0%
Febrile neutropenia	8	6 (75%) vs. 6 (75%)	0%	6 (75%) vs. 6 (75%)	0%	7 (87.5%) vs. 7 (87.5%)	0%	8 (100%) vs. 8 (100%)	0%	6 (75%) vs. 6 (75%)	0%
Hospital acquired pneumonia	7	3 (42.9%) vs. 3 (42.9%)	0%	3 (42.9%) vs. 4 (57.1%)	0%	2 (28.6%) vs. 2 (28.6%)	0%	1 (14.3%) vs. 3 (42.9%)	−28.6%	4 (57.1%) vs. 3 (42.9%)	14.3%
Pyelonephritis	8	5 (62.5%) vs. 6 (75%)	−12.5%	3 (37.5%) vs. 7 (87.5%)	−12.5%	4 (50%) vs. 4 (50%)	0%	3 (37.5%) vs. 6 (75%)	−37.5%	4 (50%) vs. 6 (75%)	−25%
Skin soft tissue infection II	7	6 (85.7%) vs. 4 (57.1%)	28.60%	7 (100%) vs. 7 (100%)	28.6%	5 (71.4%) vs. 5 (71.4%)	0%	6 (85.7%) vs. 4 (57.1%)	28.6%	6 (85.7%) vs. 5 (71.4%)	14%
Vertebral osteomyelitis	10	7 (70%) vs. 5 (50%)	20%	6 (60%) vs. 6 (60%)	20%	6 (60%) vs. 6 (60%)	0%	6 (60%) vs. 3 (30%)	30%	2 (20%) vs. 5 (50%)	−30%
**Severe infections**											
Bacterial meningitis	8	6 (75%) vs. 4 (50%)	25%	5 (62.5%) vs. 4 (50%)	25%	4 (50%) vs. 4 (50%)	0%	5 (62.5%) vs. 3 (37.5%)	25%	5 (62.5%) vs. 3 (37.5%)	25%
HIV	9	7 (77.8%) vs. 7 (77.8%)	0%	6 (66.7%) vs. 6 (66.7%)	0%	6 (66.7%) vs. 7 (77.8%)	−11.1%	6 (66.7%) vs. 6 (66.7%)	0%	6 (66.7%) vs. 7 (77.8%)	−11.1%
Infective endocarditis	9	2 (22.2%) vs. 2 (22.2%)	0%	2 (22.2%) vs. 2 (22.2%)	0%	2 (22.2%) vs. 2 (22.2%)	0%	2 (22.2%) vs. 2 (22.2%)	0%	2 (22.2%) vs. 2 (22.2%)	0%
Necrotizing fasciitis	9	8 (88.9%) vs. 6 (66.7%)	22.20%	7 (77.8%) vs. 7 (77.8%)	22.2%	6 (66.7%) vs. 6 (66.7%)	0%	7 (77.8%) vs. 6 (66.7%)	11.1%	7 (77.8%) vs. 7 (77.8%)	0%
Prosthetic joint infection	7	5 (71.4%) vs. 4 (57.1%)	14.30%	5 (71.4%) vs. 5 (71.4%)	14.3%	6 (85.7%) vs. 6 (85.7%)	0%	5 (71.4%) vs. 4 (57.1%)	14.3%	6 (85.7%) vs. 6 (85.7%)	0%
Sepsis	7	5 (71.4%) vs. 5 (71.4%)	0%	5 (71.4%) vs. 4 (57.1%)	0%	7 (100%) vs. 5 (71.4%)	28.6%	7 (100%) vs. 7 (100%)	0%	6 (85.7%) vs. 6 (85.7%)	0%
Viral encephalitis	7	3 (42.9%) vs. 1 (14.3%)	28.60%	2 (28.6%) vs. 1 (14.3%)	28.6%	1 (14.3%) vs. 1 (14.3%)	0%	2 (28.6%) vs. 2 (28.6%)	0%	2 (28.6%) vs. 3 (42.9%)	−14.3%

### 3.4 Statistical comparison of ChatGPT vs. other AI models across clinical question types

Table 5 presents the statistical significance of performance differences between ChatGPT 3.5 and the other AI platforms, as determined by chi-square or Fisher's exact tests, along with odds ratios and 95% confidence intervals to quantify effect sizes. For overall performance across all 160 MCQs, no statistically significant differences were found between ChatGPT 3.5 vs. Perplexity AI (*p* = 0.72, OR = 1.12, 95% CI: 0.71–1.76), ChatGPT 3.5 vs. Gemini (*p* = 0.24, OR = 1.33, 95% CI: 0.86–2.07), ChatGPT 3.5 vs. Copilot (*p* = 0.41, OR = 1.24, 95% CI: 0.80–1.92), or ChatGPT 3.5 vs. Meta AI (*p* = 0.36, OR = 1.27, 95% CI: 0.82–1.97). Similarly, no significant differences were observed across clinical question categories, with odds ratios indicating small effect sizes and wide confidence intervals reflecting variability in performance.

**Table 5:** Statistical comparison of AI performance: ChatGPT vs. other AI models across clinical question types.

**Category**	**No. of questions**	**ChatGPT vs. Perplexity AI**	**OR (95% CI)**	***P*-value**	**ChatGPT vs. Gemini**	**OR (95% CI)**	***P*-value**	**ChatGPT vs. Copilot**	**OR (95% CI)**	***P*-value**	**ChatGPT vs. Meta AI**	**OR (95% CI)**	***P*-value**
Overall	160	105 (65.6%) vs. 101 (63.1%)	1.12 (0.71–1.76)	0.72	105 (65.6%) vs. 94 (58.8%)	1.33 (0.86–2.07)	0.24	105 (65.6%) vs. 97 (60.6%)	1.24 (0.80–1.92)	0.41	105 (65.6%) vs. 96 (60.0%)	1.27 (0.82–1.97)	0.36
Symptoms	17	13 (76.5%) vs. 13 (76.5%)	1.00 (0.20–4.88)	1.00	13 (76.5%) vs. 12 (70.6%)	1.33 (0.28–6.37)	1.00	13 (76.5%) vs. 13 (76.5%)	1.00 (0.20–4.88)	1.00	13 (76.5%) vs. 13 (76.5%)	1.00 (0.20–4.88)	1.00
Diagnosis	29	23 (79.3%) vs. 20 (68.9%)	1.73 (0.52–5.70)	0.39	23 (79.3%) vs. 18 (62.1%)	2.34 (0.73–7.53)	0.19	23 (79.3%) vs. 18 (62.1%)	2.34 (0.73–7.53)	0.19	23 (79.3%) vs. 17 (58.6%)	2.70 (0.84–8.65)	0.13
Microorganism	21	14 (66.7%) vs. 14 (66.7%)	1.00 (0.29–3.47)	1.00	14 (66.7%) vs. 13 (61.9%)	1.23 (0.36–4.23)	1.00	14 (66.7%) vs. 13 (61.9%)	1.23 (0.36–4.23)	1.00	14 (66.7%) vs. 13 (61.9%)	1.23 (0.36–4.23)	1.00
Therapy	63	36 (57.1%) vs. 33 (52.4%)	1.21 (0.62–2.35)	0.58	36 (57.1%) vs. 34 (54.0%)	1.13 (0.58–2.20)	0.72	36 (57.1%) vs. 36 (57.1%)	1.00 (0.51–1.95)	1.00	36 (57.1%) vs. 35 (55.6%)	1.06 (0.55–2.06)	0.86
Preventive therapy	8	6 (75%) vs. 6 (75%)	1.00 (0.14–7.14)	1.00	6 (75%) vs. 5 (62.5%)	1.80 (0.25–12.86)	0.62	6 (75%) vs. 5 (62.5%)	1.80 (0.25–12.86)	0.62	6 (75%) vs. 4 (50%)	3.00 (0.46–19.68)	0.37
Risk factor	8	6 (75%) vs. 6 (75%)	1.00 (0.14–7.14)	1.00	6 (75%) vs. 5 (62.5%)	1.80 (0.25–12.86)	0.62	6 (75%) vs. 5 (62.5%)	1.80 (0.25–12.86)	0.62	6 (75%) vs. 7 (87.5%)	0.43 (0.04–4.53)	0.61
Other	14	7 (50%) vs. 9 (64.3%)	0.56 (0.15–2.06)	0.49	7 (50%) vs. 7 (50%)	1.00 (0.27–3.67)	1.00	7 (50%) vs. 7 (50%)	1.00 (0.27–3.67)	1.00	7 (50%) vs. 7 (50%)	1.00 (0.27–3.67)	1.00

### 3.5 Evaluation of AI capabilities in medical knowledge and decision support

Table 6 provides a qualitative comparison of the key system features and capabilities of the five AI platforms, complementing the quantitative performance data presented in the previous tables. All platforms possess foundational capabilities such as understanding medical language, generating general diagnostic suggestions, and offering standard evidence-based recommendations based on pre-trained data. However, notable differences emerge in areas such as real-time access to medical databases and advanced NLP algorithms. For instance, Microsoft Edge Copilot uniquely offers real-time access to clinical databases like UpToDate and PubMed, along with enhanced NLP for analyzing clinical records, setting it apart from the other systems that rely solely on pre-trained data. ChatGPT 3.5, Perplexity AI, and Meta AI pre-trained knowledge is fixed at data points from September 2021 to 2022, whereas Google Bard and Microsoft Edge Copilot use a continuously updated data system.

**Table 6:** Comparison of AI models in clinical knowledge, decision support, and data accessibility.

	**Variables**	**CHATGPT**	**Perplexity AI**	**Google bard (Gemini)**	**Microsoft edge COPILOT**	**Meta AI**
1.	Medical knowledge databases					
a	Based on pre-trained data, may include common clinical knowledge	✓	✓	✓	✓	✓
b	Real-time access to medical databases like UpToDate, PubMed	✗	✗	✗	✓	✗
2.	Natural language processing (NLP)					
a	Understanding case questions, interpreting medical language	✓	✓	✓	✓	✓
b	In-depth NLP algorithms for analyzing clinical records or real-time input	✗	✗	Limited/developing	✓	✗
3.	Diagnostic decision support					
a	General diagnostic suggestions based on symptoms and known conditions	✓	✓	✓	✓	✓
b	Real-time diagnostic algorithms based on lab results or clinical data	✗	✗	✗	✓	✗
4.	Clinical reasoning models					
a	Using learned patterns to suggest diagnoses or treatments	✓	✓	✓	✓	✓
b	Actual clinical reasoning or judgment based on direct patient interaction	✗	✗	✗	✗	✗
5.	Clinical decision support systems (CDSS)					
a	No integration with live clinical decision support systems	✓	✓	✓	✓	✓
b	Real-time access to clinical decision support for recommendations	✗	✗	✗	✗	✗
6.	Patient management guidelines					
a	General guideline-based suggestions based on pre-trained data	✓	✓	✓	✓	✓
b	No access to the latest, dynamic, or local management guidelines	✗	✗	✗	✗	✗
7.	Pathogen identification					
a	Common pathogen identification based on symptoms and lab results	✓	✓	✓	✓	✓
b	Direct pathogen identification from lab tests like Gram stains or cultures	✗	✗	✗	✗	✗
8.	Pharmacological databases					
a	Pharmacological knowledge based on common drugs and therapies	✓	✓	✓	✓	✓
b	No real-time access to updated pharmacological databases or drug interactions					
9.	Treatment duration recommendations					
a	Based on standard recommendations for certain diseases	✓	✓	✓	✓	✓
b	Dynamic, personalized treatment duration based on patient-specific data	✗	✗	✗	✗	✗
10.	Evidence-based guidelines on pharmacotherapy					
a	Standard evidence-based recommendations based on pre-existing knowledge	✓	✓	✓	✓	✓
b	No access to live, region-specific or updated evidence-based guidelines	✗	✗	✗	✗	✗
	DATA access status (Feb–Mar 2025)	Pre-trained (up to late 2024)	Real-time web data	Continuous updates via Gemini	Continuous updates via Bing and clinical databases	Pre-trained (up to early 2025)

## 4 Discussion

The research team conducted analysis to test the accuracy and reliability of five AI systems, namely, ChatGPT 3.5, Google Bard, Perplexity AI, Microsoft Copilot, and Meta AI at answering MCQs in the infectious diseases and pharmacotherapy disciplines. Microsoft Copilot provided steady results, while ChatGPT proved to be the most accurate of all systems tested, but its results had medium variability. The main strength of the study is reproducible responses, which researchers have not paid enough attention to in the past. For domain specific questions, pharmacotherapy based and microorganism identification tests were used to assess medical education and clinical decision making reliability. This work provides useful information for the distribution of clinical knowledge in the contemporary AI technology.

ChatGPT proved to have the best success rate of 65.6% in answering correctly questions about infectious diseases and pharmacotherapy, compared to other AI platforms. Success rates varied depending on question difficulty. Further research by Meo et al. (20) indicated ChatGPT achieved a 56.6% success rate, highlighting its limited understanding of public health and infectious disease topics. Fernandes et al. (12) assessed ChatGPT-3.5 and ChatGPT-4 using infectious disease specialist certification exam questions, finding ChatGPT-3.5 achieved 53.95% accuracy and ChatGPT-4 achieved 73.68% accuracy. Comparative studies with human experts further demonstrate ChatGPT's potential in medical education, though its performance in specialization exams often falls short of medical faculty graduates and students, underscoring the need for human oversight in educational settings (21, 22). Additionally, Si et al. (23) reported that ChatGPT accurately identified 74% of diseases but prescribed correct treatments only 58% of the time when managing infectious disease cases.

The performance levels of ChatGPT vary between different fields of pharmacotherapy. Wei et al. (24) analyzed ChatGPT's ability to provide pediatric pharmacotherapy recommendations, finding an accuracy of 82.2% in common diseases but lower accuracy in complex medication dosing, selection, and treatment individualization.

In our study, Perplexity AI achieved an overall accuracy of 63.2%, positioning itself just below ChatGPT (65.6%) but ahead of Google Bard (58.8%). Its median correctness rate (69.1%) and standard deviation (23.6%) suggest that Perplexity AI maintains a reasonable level of consistency. When breaking down its performance across different clinical content domains (Table 3), we observed notable variations in accuracy depending on the question type. Perplexity AI demonstrated strong performance in symptom identification (76.5%) and microorganism-based questions (66.7%), but its accuracy declined in therapy-related MCQs (52.4%) and preventive therapy questions (75%). The research findings support existing literature because Perplexity AI shows excellence in structured medical knowledge retrieval yet faces restrictions in pharmacotherapy decision-making. Research on AI medical decision systems showed Perplexity AI achieved strong performance when identifying disease symptoms yet failed to recommend proper pharmacotherapies because it lacked access to current drug databases (13). The dataset shows that Perplexity AI correctly answered 33 out of 63 therapy-based MCQs for a 52.4% accuracy rate, as it lacks detailed capabilities in medication selection, dosing, and treatment individualization. Research on AI pharmacotherapy recommendations for infectious diseases showed Perplexity AI achieved drug selection accuracy in 54% of cases, while ChatGPT performed better with 69% accuracy (14). The accuracy rate of Perplexity AI reached 71.3% when making structured pharmacotherapy recommendations, according to vascular medicine research, which supports our findings that show better performance in structured questions about symptoms (76.5%) and microorganism identification (66.7%), but lower accuracy in therapy selection (52.4%) (25). The evidence indicates that Perplexity AI has certain restrictions when it comes to customizing drug regimens and making antimicrobial choices based on evidence. The AI tool demonstrates excellence in retrieving structured medical data, but it fails to provide real-time clinical support, which limits its effectiveness as an AI model for pharmacotherapy education.

Google Bard (Gemini) ranked third in accuracy (58.8%), with a high error rate in pharmacotherapy-related MCQs (46%), correctly answering only 34 out of 63 therapy questions (53.9%). These findings align with previous studies highlighting Bard's inconsistencies in drug-related recommendations and pharmacotherapy safety assessments (26). A comparative study on drug–drug interactions (DDIs) found that Bard identified only 68 interactions compared to Lexicomp's 90, with poor agreement (κ = 0.01) in risk rating, indicating weak reliability in pharmacotherapy safety (27). Similarly, Bard's accuracy in microorganism identification (61.9%) and preventive therapy recommendations (62.5%) showed gaps in pharmacotherapy-based responses. Although a contradictory study in gynecologic oncology found Bard had an 87.5% accuracy, it still struggled with medication-based inquiries (28). Likewise, a nursing competency exam study found that Bard's score (75%) was lower than Microsoft Copilot (84%) and ChatGPT (77%), with notable weaknesses in pharmacotherapy questions (29). Despite moderate proficiency in diagnostic reasoning (62.1%) and symptom-based queries (70.6%), Bard's limitations in therapy-based MCQs (53.9%) and drug regimen selection highlight its unreliability in clinical pharmacotherapy decision-making. While useful for general medical knowledge, human oversight is essential for its application in pharmacotherapy.

The performance disparities among the AI models may reflect differences in data update mechanisms. ChatGPT 3.5, with fixed pre-trained data (cutoff late 2024), achieved 65.6% accuracy, potentially benefiting from consistent pattern recognition, while Google Bard (Gemini 1.5 Pro) and Microsoft Copilot, with real-time access, scored 58.8 and 60.9%, respectively. Bard's underperformance, despite up-to-date data, suggests challenges in synthesizing real-time information for nuanced medical reasoning (e.g., 61.9% in microorganism identification). This highlights that real-time access may not always enhance accuracy in standardized MCQs, possibly due to data integration issues, as noted in recent studies comparing Gemini and ChatGPT in clinical tasks. Future AI designs might benefit from hybrid approaches balancing fixed and dynamic data (30, 31).

Microsoft Edge Copilot demonstrated 60.9% success in pharmacotherapy MCQs during our evaluation, which placed it in fourth position among the five tested AI models. The 63 therapy-based MCQs yielded 36 correct answers (57.1% accuracy) but contained 27 incorrect responses (42.8% error rate). The combination of symptom recognition (76.5%) and microorganism identification (61.9%) was reasonable; however, its diagnostic accuracy (62.1%) and performance on preventive therapy-related questions (62.5%) demonstrated significant weaknesses, raising doubts about its clinical relevance for pharmacotherapy recommendations. In the study conducted by Fabijan et al. (32), the classification accuracy of Microsoft Copilot and ChatGPT in scoliosis treatment decision making was investigated and the results were satisfactory (32). While Copilot's responses were not as sophisticated as ChatGPT-4's, it can generate general treatment guidelines but cannot generate the complex reasoning needed to make individualized pharmacotherapy decisions. Microsoft Copilot could retrieve the correct treatment guidelines in research on chronic obstructive pulmonary disease pharmacologic management, but the guidelines were too basic for clinical use. Evidence based materials were found to be better than the recommendations from this source as they had individualized treatment plans (33). Our study results were consistent with preventive therapy performance, as Copilot achieved a 62.5% accuracy rate, but did not perform well in selecting detailed pharmacotherapy based selections, at a 57.1% success rate. The research by Ermis et al. (34) demonstrates that Microsoft Copilot delivers superior performance compared to ChatGPT in structured treatment protocols. The research showed that Copilot generated treatment recommendations for retinopathy of prematurity that were clearer, followed established guidelines, and displayed proper structure compared to responses from ChatGPT-4. The evidence shows that Copilot delivers competent structured therapeutic guidance while requiring additional improvement for customizing patient treatments (34).

The therapy-related MCQs were answered correctly by Meta AI in 35 out of 63 cases (55.6%), placing it as the lowest performing model next to Google Bard (53.9%). The decision-making process for pharmacotherapy shows a major weakness in selecting drug regimens, antimicrobial stewardship, and personalized treatment recommendations, since the model produced incorrect responses in 44.4% of cases. Previous studies have confirmed that Meta AI shows unstable performance when making clinical pharmacotherapy recommendations. The diagnostic segment of Meta AI's performance proved subpar as it scored 58.6% accuracy (17/29), whereas ChatGPT reached 79.1% and Perplexity AI achieved 69%. This lower diagnostic performance indicates that incorrect medical diagnoses may subsequently result in inaccurate treatment decisions. The research results match the findings of Alterovitz et al. (35), who discovered that Meta AI demonstrated inferior pathogen-specific treatment selection accuracy compared to GPT-4 (35). The risk factor assessment capabilities of Meta AI reached an accuracy level of 87.5% (7/8), surpassing both ChatGPT and Perplexity AI, which achieved 75% accuracy. The research by Langford et al. (14) through a meta-analysis confirmed that Meta AI demonstrates high effectiveness in identifying risk factors and general disease predispositions despite its limited ability to select appropriate therapies (14). According to Tsai et al. (36), the pharmacotherapy capabilities of Meta AI displayed conflicting data points, because it produced correct general treatment plans at a 72% rate, yet its performance diminished substantially to 49% after incorporating patient-specific data (36). This suggests that while Meta AI is capable of generating standard treatment guidelines, it lacks the adaptive reasoning necessary for customized, patient-specific therapy decisions.

A key strength of our study is its focus on AI response consistency in infectious disease MCQs—an area that has received limited prior investigation. While previous studies have examined AI accuracy in single-response settings, few have explored whether AI models maintain their accuracy over repeated queries. Given that clinical decision-making relies not just on accuracy but also on consistency, our study provides novel insights into the reproducibility of AI-generated medical knowledge. Our findings highlight varying degrees of response stability across AI platforms. Microsoft Copilot exhibited the highest consistency, maintaining identical scores (97/160; 60.6%) in both rounds. Perplexity AI also demonstrated strong reliability, with only a minor decrease from 101/160 (63.1%) to 100/160 (62.5%). Google Bard (Gemini) displayed moderate fluctuation, dropping from 94/160 (58.8%) to 93/160 (58.1%), while ChatGPT showed the most notable decline, decreasing from 105/160 (65.6%) to 93/160 (58.1%), marking a 7.5% drop in accuracy. It may be attributable due to many factors such as periodic model updates, stochastic output variability, and differences in prompt interpretation. These factors could account for fluctuations in performance and highlight the importance of ongoing benchmarking in ChatGPT. Interestingly, Meta AI was the only model to improve slightly in the second round, increasing from 96/160 (60.0%) to 97/160 (60.6%). While this improvement is minor, it contrasts with other AI platforms that exhibited slight reductions in performance. These findings align with prior research on AI response consistency. Zhou and Duan (15) found that large language AI models exhibited variability in repeated medical MCQ testing, often due to differences in how the AI interpreted prompts across multiple attempts. Similarly, Allibhai et al. (37) reported that ChatGPT-4 had a response stability of 95.7% in oncological assessments, a slightly better consistency rate than observed in our infectious disease pharmacotherapy evaluation.

The research analyzed how ChatGPT performed against other AI models in resolving 160 clinical MCQs about pharmacotherapy and infectious diseases while conducting detailed statistical comparisons between them. The performance of ChatGPT exceeded that of Perplexity AI in diagnosis-based MCQs, where it answered 23 out of 29 questions correctly (79.1%), while Perplexity achieved 20 correct answers (69.0%). This difference between the models was not statistically significant (*p* = 0.55). The performance of both ChatGPT and Perplexity AI was identical in microorganism identification questions, where they correctly answered 14 out of 21 (66.7%) questions (*p* = ns). The study conducted by Alterovitz et al. (35) demonstrated that AI models deliver relevant medical suggestions in infectious disease modeling, yet their reliability remains unstable because of reasoning and contextual integration boundaries. Large language AI model testing for tropical and infectious disease classification revealed that AI achieved the same accuracy as expert humans, yet unpredictable model responses prevent their clinical release by doctors (38). The accuracy levels between ChatGPT and Microsoft Copilot were equal at 36/63 (57.1%) for therapy-related MCQs, but ChatGPT exhibited slightly higher accuracy than Google Bard's 34/63 (53.9%), Perplexity AI's 33/63 (52.4%), and Meta AI's 35/63 (55.6%). Statistical significance was not established for these tests based on the obtained *p*-values (*p* > 0.05). The research by Jawanpuria et al. (39) showed that AI models produced satisfactory responses to infectious disease prevention and control questions with 63.6% accuracy, but provided non-specific treatment recommendations. The GPT-4 model produced plausible differential diagnoses, but research by Mondal et al. (40) showed that no AI model achieved statistical agreement with expert-generated answers during infectious disease differential diagnosis.

The research evaluated AI capabilities through MCQs from medical textbooks, but these questions might not represent the clinical challenges that medical practitioners encounter in actual practice and might not reflect the variability of real-world clinical scenarios. Therefore, these findings may not be fully generalizable to clinical practices of infectious disease specialist, where patient presentations are often more diverse and nuanced. Future studies should incorporate real or simulated clinical cases to enhance external validity or expert assessments to provide stronger validation of AI performance. And these AI tools could assist medical students in practicing diagnostic reasoning and provide a supplementary resource for self-assessment during exam preparation for specialization. While statistical accuracy was evaluated in this study, but qualitative reasoning, clarity of AI explanations, and practical usefulness of the answers in real-world data remained unassessed. Future studies should analyze AI's long-term reliability, perform qualitative response analysis, and include direct comparisons with medical experts to improve future uses of AI in clinical education and decision-making support using integrated qualitative and quantitative studies. Further research studies should focus on prospective clinical trials to validate findings in real-world clinical settings, comparative studies using real patient data to assess clinical relevance and generalizability, and longitudinal evaluations to determine the stability and consistency of AI responses over time. Additionally, the sample size of 160 MCQs may limit the statistical power to detect significant differences in AI performance, underscoring the need for a broader set of questions to enhance the robustness of statistical analyses and better reflect the diversity of clinical scenarios.

## Data Availability

The raw data supporting the conclusions of this article will be made available by the authors, without undue reservation.
